# Medicine

**Published:** 1903-12

**Authors:** J. Michell Clarke


### Progress of the Medical Sciences.

### MEDICINE.

**The "Status Lymphaticus."**—Dr. G. Blumer discusses the connection of this condition with sudden death occurring spontaneously, under anæsthesia, and during infectious disease.[1] These cases are of interest from both a professional and medico-legal point of view. From the former it is most important to be able to recognize the subjects of a peculiar constitution which renders them extremely susceptible to shocks of various kinds, physical, psychical, and toxic, and which may lead to their sudden death from an accident which to an ordinary individual would be trivial; *e.g.*, death from the shock of a plunge into cold water, or, in the case of Prof. Langerhans's child, a few minutes after the administration of antitoxin. A suspicion of foul play may be entertained in cases of sudden death in apparently healthy infants, and cases are recorded where nurses or relatives have been accused of suffocating or maltreating infants, the true cause of death being shown at the autopsy to be the status lymphaticus.

Is it possible to make a diagnosis before the fatal attack? Escherich states that these cases usually have a pale, thin skin, a pasty complexion, and a good pad of subcutaneous fat. Frequently signs of rickets or scrofula are present. Superficial lymph-glands, especially of the neck and axilla, are enlarged. There is hypertrophy of tonsils, and of circumvallate papillæ of tongue, and adenoids. The spleen can often be felt; a percussible thymus may be developed, and Ewing's work suggests that the lymphocytes are increased. Unfortunately, as these patients are mostly in apparent good health until within a short time of death, the knowledge of clinical symptoms mainly relates only to that period. The immediate causes of death are two: cardiac paralysis and asphyxia.

Clinically the cases comprise:—(1) Those found dead in bed. These are mostly young infants; probably many cases of "overlying" are really due to this cause; a death in convulsions is suggested by the history of some of them. (2) Those of sudden death, presumably of cardiac origin. In all probability, cases reported by Nordmann and others, where sudden immersion in cold water was followed by instant death, belong to this class. (3) Those of sudden death, presumably of respiratory origin. Most of these cases may be classed under "thymic asthma." This condition has been confounded with ordinary

1 "The Relation of the Status Lymphaticus to Sudden Death, Death under Anæsthesia and Infection." *Johns Hopkins Hosp. Bull.*, 1903, xiv. 270.

laryngismus stridulus, but there is probably no relation between them. Cases of thymic asthma vary in clinical type; sometimes the child dies in the first attack or series of attacks, or the condition may last for a week or even years, and finally terminate fatally or be relieved by operative extirpation of the thymus. In some cases there are no definite asthmatic seizures; the child presents accelerated respiration, an access of suffocation, cyanosis, dilated pupils, swelling of the veins in the neck, and death occurs in a few minutes. (4) Those of sudden death, presumably of nervous origin. Occasionally the status lymphaticus is associated with epilepsy. Some cases have died very suddenly during a spasm described as epileptiform. An unusual, so-called cerebral form of death from status lymphaticus has been described by Laub. It usually occurs in young adults, and causes death in a few hours, eighteen at the outside. The patient is attacked suddenly with coma, or perhaps epileptiform convulsions; sometimes there is spasm of the glottis or vomiting of a cerebral type; the urine contains albumin, but no casts. The *post-mortem* shows the presence of cerebral œdema, together with the ordinary signs of status lymphaticus.

**Death during Anæsthesia.**—Within the last few years attention has been called to the relation of the status lymphaticus to deaths during or soon after the administration of an anæsthetic. Almost all observers agree that death is due to cardiac failure; in some cases premonitory symptoms occur, but in many it is instantaneous. At first it was thought that chloroform was especially dangerous, but in recent years death has occurred under ether and the A.C.E. mixture. It seems that two classes of patients are especially liable to this accident: those with goître, either simple or exophthalmic, and those with adenoid vegetations.

Little is known of the **relation of the status lymphaticus to infection.** The author gives a fatal case of tetanus and one of general streptococcic infection modified by the status lymphaticus, and quotes Daut's paper on cases from Escherich's clinic, who found that over 25 per cent. of patients dying from diphtheria presented the picture of this state. The symptoms were in some cases a barking cough, hoarseness, attacks of spasmodic suffocation, weakness of heart, these attacks being altogether out of proportion to the amount of membrane, and in others sudden death. It would thus seem that the status lymphaticus may have a distinct influence on infections, sometimes modifying their course and sometimes masking them.

**Pathological Anatomy.**—This consists, broadly speaking, of a hyperplasia of the thymus gland and of the lymphatic tissues. The degree and extent of this hyperplasia vary, involving sometimes a special group of glands, or the intestinal and splenic lymphatic tissue especially, or the lymphatic tissue of the body generally; in practically all cases the thymus is affected. Other lesions often present are hypoplasia of the vascular system, especially in adults; compression of trachea by enlarged thymus, in which case there may be subpleural and other subserous hemorrhages and atelectatic patches in lungs; œdema and congestion of lungs; œdema of brain; lymphoid condition of bone marrow; enlarged thyroid. Histologically, besides a general hyperplasia of lymphoid elements in thymus and in lymph nodes, Malpighian corpuscles of spleen, tonsils, and lymphoid tissue of intestine, there is a proliferation of the cells composing the germinal centres in lymph nodes, tonsils, and intestinal apparatus, associated with slight degenerative changes in the proliferated cells, and similar changes in the Malpighian bodies of the spleen.

Bacteriological investigation in the author's cases was negative.

With regard to the explanations advanced to account for sudden death in the status lymphaticus, the mechanical theory that death is due to asphyxia from compression of the trachea has met with much opposition. The author believes that the opposing views are largely due to diverse individual experience, for although many of the cases show no evidence of tracheal compression, in others the evidence is very convincing. He considers that there is no anatomical support for the theories that compression of the heart, great vessels, or the large nerves or nerve-plexuses, is the cause of death. Nor does he think that there is sufficient evidence for the view that the condition is due to an over-production of the internal secretion of the thymus. He refers to the great similarity which exists between the lesions of the status lymphaticus and those produced by Flexner in guinea-pigs by lymphotoxins, and described by him as lymphotoxæmia. He considers that the condition in the status lymphaticus is one of intermittent lymphotoxæmia, *i.e.*, that the regulatory mechanism which prevents either the action or the formation of autocytotoxins is at times in abeyance. He suggests that these patients are born with an instability of this mechanism so far as the lymphatic apparatus is concerned, so that they are subject to intermittent attacks of lymphotoxæmia. During the attacks they are especially susceptible to the action of bacterial or chemical poisons, and also to physical or psychical shocks, which may then cause their death under circumstances which would be trivial in a healthy person.

In connection with this subject we may refer to the paper read by Professor Ruhrah at the Swansea Meeting, "On the Relation of the Thymus Gland to Marasmus,"[1] in which he arrived at the following conclusions:—(1) Atrophy of the thymus gland is always found in cases of infantile atrophy. (2) The condition of the thymus is an index of the general nutrition of the infant. (3) The state of nutrition in infants may be estimated by a microscopic examination of the thymus

[1] *Brit. M J.* 1903, ii. 455

at necropsy. In the discussion which followed this paper, the question of sudden death in infants in whom a large thymus was found at the necropsy was raised, and considerable difference of opinion was expressed as to the cause of death in such cases. It was suggested that the normal thymus in a well-nourished child, such as cases of "thymus death" usually were, might be taken as enlarged because the condition of the gland in infants dying from chronic disease would be more familiar. Professor Ruhrah said there were undoubted cases of "thymus death," but he was inclined to think that some of those reported were due to other conditions, and that the normal thymus seemed unusually large because the atrophied one was more familiar. In the pathological reports of the nine cases in Dr. Blumer's paper the weight of the thymus is only given in two: it weighed 27 grammes in a child of ten months, and 30 grammes in one of seven years. The normal weight at birth is 12 grammes. The measurements given appear, except in two instances, to be in excess of the normal, sometimes very much so.

**Surgical Treatment of Brain Tumours.**—Dr. Allen Starr[1] gives the following results of operation for brain tumours:—

| | | Cerebral. | Cerebellar. |
|---|---|---|---|
| Total number of cases operated upon ... ... ... ... ... ... | 365 | 315 | 50 |
| Cases in which the tumour was not found ... ... ... ... ... | 111 | 91 | 20 |
| Cases in which the tumour was found, but not removed ... ... | 27 | 21 | 6 |
| Cases in which the tumour was removed and the patient died... | 59 | 51 | 8 |
| Cases in which the tumour was removed and the patient recovered | 168 | 152 | 16 |

Two elements which have improved the chances of success in such operations are:—(1) Accuracy of diagnosis and of localization of a tumour are to-day more easily possible than before; it can be determined with considerable accuracy at the present time, first, that a tumour is present, and, second, that the surgeon can reach it. (2) Improvement in surgical methods of access to the brain. The method at present used by McCosh is the best as yet devised. (For details the reader is referred to the original paper.) By this method the skull can be opened in from six to eight minutes after the original incision has been made through the scalp.

Further, extensive cutting of the brain and exsection of parts of it are not necessarily attended by danger, provided the hemorrhage can be arrested. For the above reasons the author thinks it probable that the statistics of the next ten years will

[1] *J. Nerv. and Ment. Dis.* 1903, xxx. 398.

show a very much greater percentage of successes than do those up to the present.

Dr. Starr considers the operation for **cerebellar** tumours to be one of great difficulty and danger. Cerebellar tumours as a rule lie either in the middle lobe of the cerebellum, immediately beneath the tentorium, or in the sulci between the cerebellum and the bulb, both of which situations are inaccessible. After a considerable experience of nine such cases, operated upon without any success, he considers it futile to attempt this operation.

**Hoppe**, in a paper on the same subject,[1] comes to the following conclusions:—(1) Cortical or subcortical tumours of any portion of the cerebral hemispheres which can be reached through the calvarium are operable. (Allen Starr remarks in the paper just quoted that whilst the difficulties of removing a deep tumour are many, and the chances of recovery, first, from the operation, and, second, from the symptoms, are much less in deeply-seated tumours, yet a growth lying one to two inches below the surface may be reached.) (2) If possible, the operation should be performed early, when the tumour is small, but even large tumours and those infiltrating in character have been operated on with success. (3) With few exceptions, the tumours hitherto successfully operated on belong to the psycho-motor area. (4) The result of surgical interference, even in the most successful cases, rarely leads to complete recovery; as a rule, symptoms due to increased intracranial pressure disappear after the operation, whilst the focal symptoms mostly remain or are only diminished. (5) Cerebellar tumours are inoperable. (6) Cumulative experience is against exploratory operations. (7) The question of palliative operations has a considerable weight of opinion on either side; the author, whilst leaving the question open at present, is personally opposed to them. (8) He thinks that gummata should be operated upon if they give the signs of tumour, and isolated tubercles also, if the conditions are otherwise favourable.

J. MICHELL CLARKE.

### SURGERY.

1 "A Report of Seven Operations for Brain Tumours and Cysts." *J. Am. M. Ass.*, 1901, xxxvi. 302.

